# Adrenergic System Activation Mediates Changes in Cardiovascular and Psychomotoric Reactions in Young Individuals after Red Bull^**©**^ Energy Drink Consumption

**DOI:** 10.1155/2015/751530

**Published:** 2015-06-01

**Authors:** Ana Cavka, Marko Stupin, Ana Panduric, Ana Plazibat, Anita Cosic, Lidija Rasic, Zeljko Debeljak, Goran Martinovic, Ines Drenjancevic

**Affiliations:** ^1^Department of Physiology and Immunology, Faculty of Medicine Osijek, Josip Juraj Strossmayer University of Osijek, 31000 Osijek, Croatia; ^2^Department of Cardiovascular Medicine, Internal Medicine Clinic, University Hospital Center Osijek, 31000 Osijek, Croatia; ^3^Department of Pharmacology, Faculty of Medicine Osijek, Josip Juraj Strossmayer University of Osijek, 31000 Osijek, Croatia; ^4^Department of Clinical Laboratory Diagnostics, University Hospital Center Osijek, 31000 Osijek, Croatia; ^5^Faculty of Electrical Engineering Osijek, Josip Juraj Strossmayer University of Osijek, 31000 Osijek, Croatia

## Abstract

*Objectives.* To assess the effect of Red Bull^©^ on (1) blood glucose and catecholamine levels, (2) cardiovascular and respiratory function changes before, during, and after exercise, (3) reaction time, (4) cognitive functions, and (5) response to mental stress test and emotions in young healthy individuals (*N*=38). *Methods.* Heart rate (HR) and arterial blood pressure (ABP), blood glucose, adrenaline, and noradrenalin plasma levels were measured before and after Red Bull^©^ intake. Participants were subjected to 4 different study protocols by randomized order, before and 30 minutes after consumption of 500 mL of Red Bull^©^. *Results.* Mean ABP and HR were significantly increased at rest after Red Bull^©^ intake. Blood glucose level and plasma catecholamine levels significantly increased after Red Bull^©^ consumption. Heart rate, respiration rate, and respiratory flow rate were significantly increased during exercise after Red Bull^©^ consumption compared to control condition. Intake of Red Bull^©^ significantly improved reaction time, performance in immediate memory test, verbal fluency, and subject's attention as well as performance in mental stress test. *Conclusion.* This study demonstrated that Red Bull^©^ has beneficial effect on some cognitive functions and effect on cardiovascular and respiratory system at rest and during exercise by increasing activity of the sympathetic nervous system.

## 1. Introduction

Although the consumption of energy drinks is somewhat declining [1, 2] compared to previous decades [3], still a lot of young people, athletes, and especially college students consume energy drinks before practicing, while studying, and before exams because they believe that this improves their performance and memory [4]. Red Bull^©^ is among the most popular energy drinks with annual global sales of several billion dollars [5]. Red Bull^©^ is sold under the slogan “gives you wings” which suggests that its consumption will provide the consumer with more energy and enhanced performance, both mentally and physically. The manufacturers credit these physiological and neurological benefits to Red Bull^©^'s active ingredients that include: caffeine (approximately 32 mg/dL), glucuronolactone (approximately 240 mg/dL), and taurine (approximately 400 mg/dL), as well as B vitamins (thiamine, riboflavin, niacin, pantothenic acid, pyridoxine hydrochloride, biotin, inositol, and cyanocobalamin) and sugars (glucose and saccharose) [6].

In terms of physiological effects of Red Bull^©^, most of the documented changes have focused on the subjects' blood pressure (BP) and heart rate (HR) at rest [7, 8]. The results of these studies are inconclusive for both initial assumed effects of the consumption of energy drink, raising the BP and lowering the HR [4, 7–9]. These contradictory results may occur due to opposing effects of two active ingredients of Red Bull^©^: caffeine and taurine. Caffeine is characterized as a central nervous system stimulant promoting activation of the sympathetic adrenal-medullar system that leads to immediately increased blood pressure and peripheral vascular resistance [6, 10, 11]. On the other hand, taurine, a sulfur-containing amino acid, seems to suppress sympathetic nervous system stimulation by modulating cyclic nucleotide content in heart cells [12, 13]. Limited number of controlled studies on Red Bull^©^ effects on exercise performance has given inconsistent results, as well. Most of the studies on young athletes indicate that energy drinks improve endurance performance [14], although there is less support for its ergogenic properties during strength- and power-based exercise dependent upon oxygen-independent metabolism [15, 16]. Observed ergogenic benefits of energy drinks are likely attributable to caffeine and glucose content [6, 17]. The notable findings of some recent studies are that nowadays caffeine content in energy drinks is comparable to coffee beverages in Europe and USA [1, 18].

Even though Red Bull^©^ is promoted as energy drink that improves mental alertness and performance, the number of studies that researched this topic is limited. Alford et al. found that Red Bull^©^ improved reaction times, alertness, and concentration and that improved performance on an immediate recall memory test in young healthy students [7]. The other study has shown that Red Bull^©^ has beneficial effect on cognitive performance and mood in graduate student population [19]. On contrary, Bichler et al. demonstrated that Red Bull^©^ energy drink has no effect on short term memory in young college students [4]. The results of one recent study indicated that energy drink consumption decreased reaction times on behavioral control tasks, increased subjective ratings of stimulation and decreased ratings of mental fatigue [20]. The number of young people taking energy drinks is still high, and the results of studies that have investigated the effects of Red Bull^©^ intake and the possible role of adrenergic system activation and other mechanisms through which its active ingredients work are inconsistent.

Since activation of sympathoadrenergic system is crucial in cardiovascular and psychomotoric reactions and Red Bull^©^ has ingredients that could modulate levels of adrenergic hormones, the aim of our study was to determine the involvement of adrenergic system in effects contributed to Red Bull^©^ consumption on (1) HR, BP, blood glucose, and plasma catecholamines levels at rest, (2) cardiac, and respiratory changes before, during, and after moderate exercise, (3) reaction time to audio stimuli, (4) cognitive functions, and also (5) response to mental stress (distracting three digit numbers) test and emotions in young healthy individuals.

## 2. Methods

### 2.1. Study Population

Thirty-eight young healthy medical students were recruited for the study by advertisement at the Faculty of Medicine Josip Juraj Strossmayer, University of Osijek. All volunteers were self-described as healthy, with no history of cardiovascular, urinary, digestive, or metabolic diseases. All subjects were familiar with Red Bull^©^ and most of them had previously consumed this energy drink. Written informed consent was obtained from each subject. The study protocol and procedures conformed to the standards set by the latest revision of the* Declaration of Helsinki* and were approved by the Ethical Committee of Faculty of Medicine, University of Osijek.

### 2.2. Study Protocol

All subjects were instructed not to consume alcohol or energy drinks 7 days prior testing. They all were fasting 12 h prior to the onset of the experiment. Subject height and weight were measured to determine body mass index (BMI), as well as the extent of hips and waist to determine waist-to-hip ratio (WHR). BP and HR were measured at the beginning of the experiment after 15 minutes rest in seated position. Semiautomatic oscillometric monitor (OMRON) was used. Final values of BP and HR were mean of three repeated measurements. Intravenous cannula was inserted into a vein and a venous blood sample was taken after 30-min resting in supine position. A separate group of 9 participants stay resting in supine position during whole study visit and blood samples for plasma catecholamine levels were taken after 30 minutes resting in supine position (before Red Bull^©^ consumption) and 15, 30, 45, and 60 minutes after 500 mL of Red Bull^©^ consumption. This was necessary due to great variability in catecholamine levels when person in moving and results of the measurements would be inconclusive for the observed effects. Prior to Red Bull^©^ consumption, other 29 participant were assigned to 4 different study protocols (described below) by randomized order. After control protocol subjects were instructed to drink 500 mL of Red Bull^©^ and after 30 minutes of resting in supine position, all tests, including BP, HR, blood tests, and each of 4 study protocols were repeated by randomized order.

#### 2.2.1. Protocol 1: Influence of Red Bull^©^ Consumption on Cardiac and Respiratory Changes during Moderate Exercise

The Harvard Step Test (HST) was used to detect cardiac and respiratory changes during moderate exercise. The HST is performed in a manner that the subject steps up and down into the default beat for three minutes on the 45 centimeters high step bench. Male participants had stepped on the bench 24 times in one minute (24/per min), while female participants had stepped on the bench 22 times in one minute (22/per min). For determining the rate, metronome was used and was set at 96 beats per minute for male subjects and 88 beats per minute for female subjects. Each tick marked a movement with subject's leg. At the START signal participant stepped up, first with one foot on the bench, and then another, and after stretching legs and straightening back immediately stepped down, gliding with leg which first stepped up. Stepping up and down always started with the same leg (with maximum two changes during the test). In one hand subject held air flow transducer and temperature sensor was attached to his other hand. The physiological functions of the subject were measured during seven minutes, first minute at rest, next three minutes during stepping up and down, and last three minutes at rest after exercise.

Three parameters were measured during this study protocol: HR, air flow, and skin temperature (ST). These parameters were assessed with three electrodes attached to subject's chest, air flow transducer into which subject breathed with clamp on his nose and skin temperature sensor mounted on his finger which were all connected to BIOPAC device (BIOPAC Systems Inc., Goleta, CA, USA). For data processing BIOPAC software was used (BIOPAC Systems Inc., Goleta, CA, USA). All measurements were performed by a single trained operator.

#### 2.2.2. Protocol 2: Influence of Red Bull^©^ Consumption on Reaction Time

Reaction time (RT) was measured by BIOPAC device and software (BIOPAC Systems Inc., Goleta, CA, USA). Measurement was performed in quiet room. Subject was sitting eyes closed with headphones and with push-button device in his hand which he pressed on sound signal. The first part of the test consisted of series of ten beeps that occurred in randomized time intervals, and second part in which beeps occurred in fixed time intervals. Both tests lasted for one minute, with one minute pause between tests.

#### 2.2.3. Protocol 3: Influence of Red Bull^©^ Consumption on Cognitive Functions

All cognitive tasks were assessed by randomized order in quiet room by a single trained operator. For cognitive function assessment following tests were used.

*Auditory Verbal Learning Test (AVLT).* To examine global cognitive functions, the alternate form of Rey's Auditory Verbal Learning Test (AVLT) published by Lezak in 1995 was utilized [21]. This cognitive task has been shown to reflect not only specific verbal learning and memory, but also global cognitive functions [22–24]. Two lists (A and B) of 15 words were used, for repeated tests parallel lists of words were available. Five presentations of the list A were given, each followed by attempted recall. The first trial of the AVLT is a measure of immediate memory. After five trials a second 15-word list (list B, distraction list) was read and followed by a recall trial of this list and then another recall trial of the list A (measure of delayed recall). After a delay interval of 30 minutes and no further presentations of the lists, delayed verbal memory was assessed by recall trial of the list A. 

*Fluency Tasks.* Each participant underwent three types of verbal fluency tests: phonemic, semantic, and ideational [21]. Each test consisted of trials (90 second duration) directing participants to generate as many words as possible (a) that began with a particular letter (“F,” “A,” or “S”) excluding proper names and variations of the same word, (b) that were exemplars of an unprimed semantic category (e.g., animals), or (c) that were exemplars of an unprimed ideational category (e.g., metal objects). The final score was given as number of correct words generated during first 60 seconds. 

*Auditory Digit Span Task (ADST).* The digit span task is one of Wechsler's group of intelligence tests used to access the immediate and working memory function [25, 26]. A list of random numbers was used and read out loud at the rate of one per second. The participants were asked to repeat an increasing array of numbers in backwards (working memory) or forward order (immediate memory) until they committed an error. 

*d2 Attention Loading Test.* This test was constructed by German psychologist Rolf Brickenkamp in 1962. We have used an updated and revised form published in 1994. The d2 attention loading test is one of the general competence tests that have been used for measuring attention and ability to concentrate, sustained attention. In the present study, an official form of the attention loading test was used (Copyright by Hogrefe, Verlag GmbH & Co., KG Göttingen, 1994). It is a timed test composed of the letters “d” and “p” with one, two, three, or four dashes arranged either individually and/or in pairs above and below the letter. There are 14 lines of 47 characters each. Subjects are given 20 seconds to scan each line and cross all the “d” marked with two dashes. The test analysis appoints quality and quantity of processed data, in general and during the time of the test. Several variables were used for the test analysis, as follows: TN: total number of processed data, E: total errors (OE: omitted errors + RE: replacing errors), TN-E: quantitative measure defined by total number of processed data minus number of total errors, MC: mean concentration, qualitative measure calculated as number of correctly processed data reduced for number of omitted errors. MC is a variable that protects from possible cheating on the test (random data processing cannot increase MC). 

#### 2.2.4. Protocol 4: Influence of Red Bull^©^ Consumption on Emotional Status and Mental Stress Response

*Mental Stress Test.* Mental stress test (MST) was used to provoke subject's emotional response. Stressor was 2-minute long arithmetic test in form of, at first sight, simple arithmetic operation (e.g., 345 – 193 = 152). Subject's task was to answer is the operation correct or incorrect with time limit of 3 seconds for each task. If subject did not offer answer, it was recorded as incorrect. After each answer, subject got feedback information if the answer was correct or not by different sound signals. In 2 minutes subject got 40 different arithmetic tasks, and after the test got information of his success. With 100% correct answers subject got 400 points. To pass the test it was necessary to respond correctly on 80% of the tasks and get 320 points. Subject was informed about his success on the test. After MST participants were subjected to Aggression Questionnaire and Beck Anxiety Scale.

*Aggression Questionnaire.* The Aggression Questionnaire AG-87 is intended to measure the tendency toward aggressive behavior in provoking situations, to measure impulsive aggression. Questionnaire A-87 consists of 15 items of different situations with five possible responses. The possible responses or reactions are the five most frequent forms of aggressive responses: (a)* verbal manifest aggression* (VM); (b)* physical manifest aggression* (PHM); (c)* indirect aggression* (IND); (d)* verbal latent aggression* (VL), and (e)* physical latent aggression* (PHL). The subject's answers were given on a five-point scale: (1) they never behave in that way, (2) they behave seldom in that way, (3) they behave in that way from time to time, (4) they behave frequently in that way, and (5) they behave very often in that way. Total test score may lie in the range between 75 and 375 points. Research has shown that the A-87 has satisfactory psychometric properties [27–29].

#### 2.2.5. Beck Anxiety Scale

The Beck Anxiety Inventory (BAI), created by Dr. Aaron T. Beck and other colleagues, is a 21-question multiple-choice self-report inventory that is used for measuring the severity of an individual's anxiety. The BAI consists of twenty-one questions about how the subject has been feeling in the last week, expressed as common symptoms of anxiety (such as numbness, hot and cold sweats, or feelings of dread). Each question has the same set of four possible answer choices, which are arranged in columns and are answered by marking the appropriate one with a cross. These are (1) not at all, (2) mildly: it did not bother me much, (3) moderately: it was very unpleasant, but I could stand it, (4) severely: I could barely stand it. In our study the version of this test with 14-question multiple-choice self-report inventory was used [30, 31].

### 2.3. Laboratory Testing

Blood samples were analyzed for blood glucose levels and plasma catecholamine levels at the Department of Clinical Laboratory Diagnostics, University Hospital Center Osijek. Adrenaline and noradrenalin levels were evaluated by using commercially available reagent kit (Catecholamines in plasma, Chromsystems, Germany) which allows the routine analysis of adrenaline and noradrenalin in plasma using an isocratic high-performance liquid chromatography (HPLC) system (Shimadzu, Japan) and an electrochemical detector (ECD, Chromsystems, Germany).

### 2.4. Statistical Analysis

All results are presented as mean ± SD. The normality of data distribution was assessed by Kolmogorov-Smirnov Normality test. Clinical characteristic between two measurements (before and after Red Bull^©^ consumption) were compared by paired *t*-test. Wilcoxon rank-sum test was used when variables were not normally distributed. To compare parameters between experimental protocols Student's *t*-test was used. When variables were not normally distributed Mann Whitney Rank Sum Test was used. To compare differences between catecholamine levels during different time periods One-Way ANOVA Repeated Measures was used. Statistical significance was set at *P* < 0.05. For statistical analysis Sigma Plot (version 11.2, Systat Software, Inc., Chicago, USA) program was used.

## 3. Results

Thirty-eight young healthy college students with a mean age of 23 ± 2 years completed the study (15 female and 23 male participants). BMI of 23.95 ± 3.03 kg/m^2^ and WHR of 0.79 ± 0.05 were measured. Mean of three BP measurements confirmed normotension before Red Bull^©^ consumption among participants.  Table 1 summarizes the values of arterial BP and HR of study population. Systolic blood pressure (SBP) was not significantly changed after Red Bull^©^ intake. However, diastolic blood pressure (DBP) was significantly increased after Red Bull^©^ consumption, compared to control measurement. Mean arterial pressure (MAP) was significantly increased 30 minutes after Red Bull^©^ consumption. There was significant increase in heart rate 30 minutes after Red Bull^©^ intake, too. All participants had normal fasting blood glucose level with no history of impaired glucose intolerance or diabetes mellitus. Blood glucose level significantly rose 30 minutes after Red Bull^©^ consumption (plasma glucose mmol/L before Red Bull^©^ consumption 4.6 ± 0.4 versus after Red Bull^©^ consumption 7.0 ± 1.4, *P* < 0.001).

### 3.1. Influence of Red Bull^©^ Consumption on Plasma Adrenaline and Noradrenalin Levels

**Figure 1:**
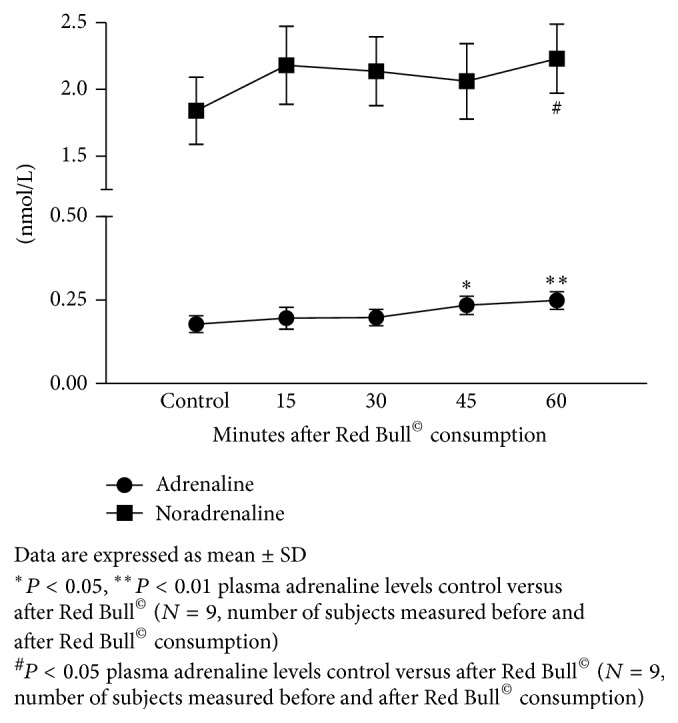
Influence of Red Bull^©^ on plasma adrenaline and noradrenalin levels.

Figure 1 summarizes influence of Red Bull^©^ consumption on plasma adrenaline and noradrenalin levels that were measured in five different time points (before Red Bull^©^ consumption and 15, 30, 45, and 60 minutes after Red Bull^©^ consumption). Both adrenaline and noradrenalin plasma levels tended to increase after Red Bull^©^ consumption in all measured time points with statistical significance for adrenaline (*P* = 0.0096) at 45 and 60 minutes time points compared to before Red Bull^©^ consumption and noradrenalin (*P* < 0.0001) at 60 minutes time points compared to before Red Bull^©^ consumption.

### 3.2. Influence of Red Bull^©^ Consumption on Cardiac and Respiratory Changes during Harvard Step Test

**Figure 2:**
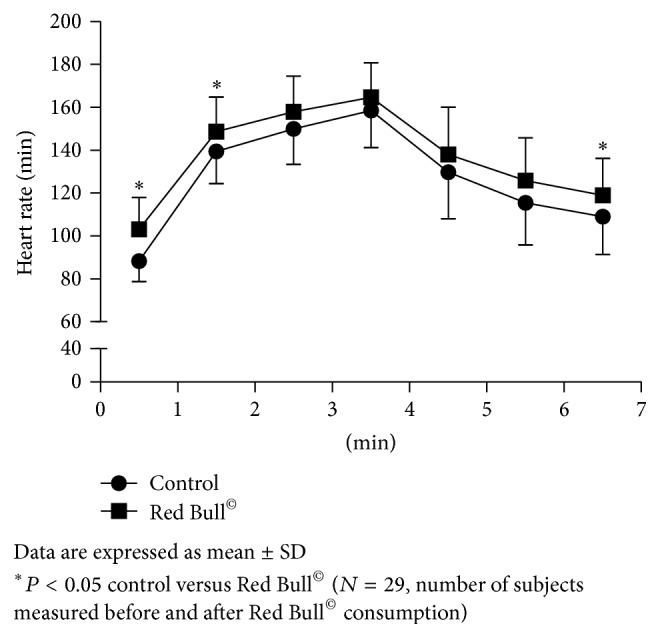
Change of heart rate (HR) during 1-minute rest, 3 minute of Harvard Step Test, and 3-minute rest after exercise before and after Red Bull^©^ consumption.

**Figure 3:**
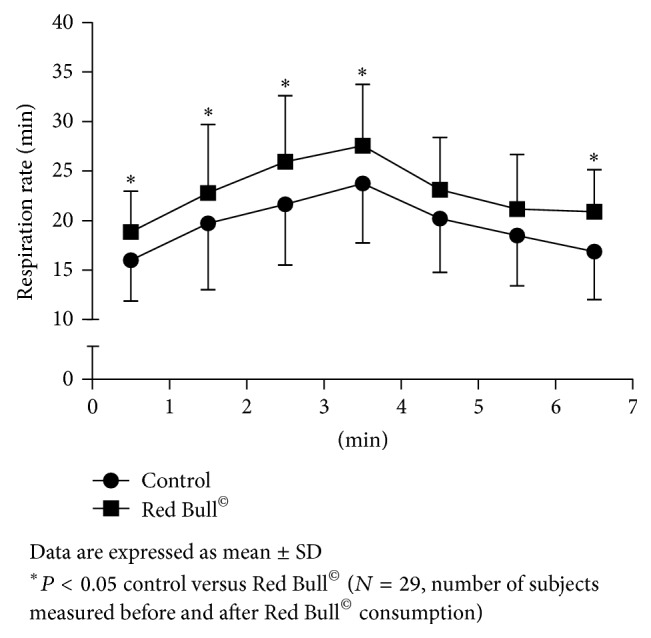
Change of respiration rate (RR) during 1 minute rest, 3 minute of Harvard Step Test and 3 minute rest after exercise before and after Red Bull^©^ consumption.

**Figure 4:**
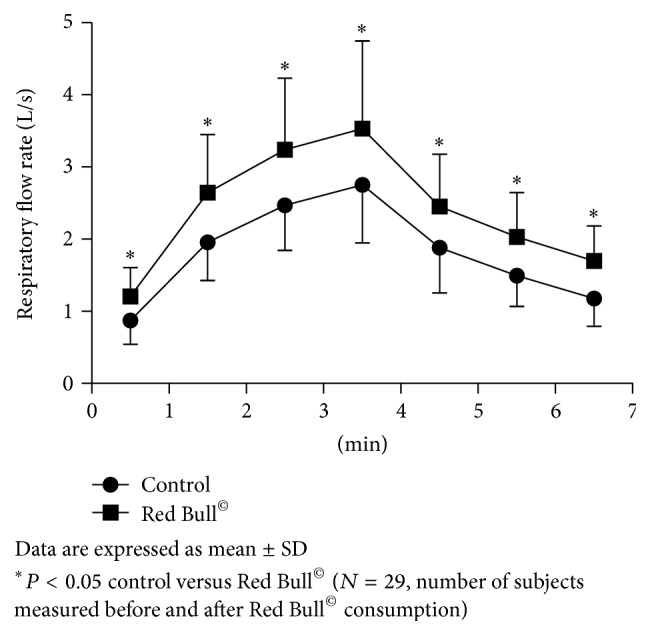
Change of respiratory flow rate (RFR) during 1-minute rest, 3-minute of Harvard Step Test, and 3-minute rest after exercise before and after Red Bull^©^ consumption.

Measurement of HR, respiration rate (RR), and respiratory flow rate (RFR) during 1-minute rest before the beginning of Harvard Step Tests (HST) has shown significant changes after Red Bull^©^ consumption. The first-minute HR during rest before the beginning of HST was significantly increased after Red Bull^©^ intake compared to control condition before Red Bull^©^ intake (HR/min 89 ± 10, 103 ± 14, resp.; *P* < 0.001). In the same condition, RR and RFR were also significantly increased after Red Bull^©^ consumption compared to control condition before Red Bull^©^ intake (RR/min 16.0 ± 4.1, 18.8 ± 4.1, *P* < 0.001; RFR L/s 0.87 ± 0.33, 1.21 ± 0.4, resp.; *P* < 0.001) (Figures 2, 3, and 4). Skin temperature (ST) was not significantly changed during rest after Red Bull^©^ intake, compared to control resting condition before Red Bull^©^ intake (ST °C 32.1 ± 0.3, 32.2 ± 0.4, resp.; *P* = 0.081).

Same parameters were measured during 3 minutes of HST before and 30 minutes after Red Bull^©^ intake. As expected, HR, RR, and RFR were increasing with the exercise duration before and after Red Bull^©^ consumption. ST did not change during 3 minutes of HST before and after Red Bull^©^ intake. HR was significantly higher (*P* < 0.001) during first minute of HST after Red Bull^©^ consumption compared to the control (Figure 2). RR and RFR were statistically significantly higher during all 3 minutes of HST after Red Bull^©^ intake compared to the control HST (Figures 3 and 4). ST did not change during exercise after Red Bull^©^ compared to the control HST.

As expected, HR, RR, and RFR were decreasing during 3 minutes rest after control HST and HST that followed Red Bull^©^ intake. Still, HR (Figure 2) and RR (Figure 3) were significantly higher during third minute at the rest after HST following Red Bull^©^ intake compared to control before Red Bull^©^ consumption (Figure 2). RFR was significantly higher during all 3 minutes at the rest after HST following Red Bull^©^ consumption, compared to the control (Figure 4). ST was increasing during all three minutes of rest after pre-Red Bull^©^ control HST and HST following Red Bull^©^ intake, but without differences between these two measurements.

### 3.3. Influence of Red Bull^©^ Consumption on Reaction Time

Reaction time (RT) was measured before and after Red Bull^©^ consumption. In both measurements, subjects reacted faster when sound signals appeared in randomized time intervals compared to the RT when sound signals appeared in fixed time intervals (RT sec before Red Bull^©^ 0.26 ± 0.04, 0.29 ± 0.04, *P* = 0.002; after Red Bull^©^ 0.24 ± 0.04, 0.27 ± 0.03, *P* < 0.001). Subjects had faster RT after Red Bull^©^ consumption compared to the control pre-consumption condition in both test, independently on sound signals appearing in fixed (*P* < 0.001) and randomized (*P* < 0.001) time intervals.

### 3.4. Influence of Red Bull^©^ Consumption on Cognitive Functions

Table 2 presents the results achieved in particular cognitive tasks. Results achieved in immediate memory (measured as number of correctly recalled words after first reading) in AVLT did not show any difference after Red Bull^©^ consumption compared to control. However, immediate memory (measured as number of correctly repeated numbers in forward order) in ADST was significantly improved after Red Bull^©^ intake compared to control. There was no significant difference in delayed recall or verbal memory measured by AVLT in control test compared to test after Red Bull^©^ intake. Working memory (measured as number of correctly repeated numbers in backward order) was significantly better after Red Bull^©^ intake. Verbal fluency (phonemic, semantic, or ideational) was significantly better after Red Bull^©^ consumption compared to control measurement. There was a significantly better performance in the d2 attention loading task (TN-E and MC) after Red Bull^©^ intake compared to the performance in control d2 attention loading task.

### 3.5. Influence of Red Bull^©^ Consumption on Emotional Status and Mental Stress Response

Participants' response to the mental stress test (MST) was significantly better after Red Bull^©^ intake compared to the control (MST points control 146.56 ± 50.73 versus points after Red Bull^©^ 189.31 ± 48.03, *P* < 0.001). However, subjects performance in Aggression Questionnaire AG-87 (AG-87 0 participants had same answers before and after Red Bull^©^ consumption, 16 participants (55.17%) had more points before Red Bull^©^ consumption and 13 participants (44.83%) had more points after Red Bull^©^ consumption; *P* = 0.502) and Beck Anxiety Inventory did not show any significant differences between two measurements, before and after Red Bull^©^ consumption (BAI 23 participants (79.13%) had same answers before and after Red Bull^©^ consumption, 4 participants (13.79%) had more points before Red Bull^©^ consumption, and 2 participants (6.9%) had more points after Red Bull^©^ consumption; *P* = 0.414).

## 4. Discussion

The salient finding of the present study is that Red Bull^©^ consumption (1) increased activity of the sympathetic nervous system (by increased plasma adrenaline and noradrenalin levels); (2) subsequently affected cardiovascular and respiratory system during rest and exercise; and (3) improved cognitive functions, as well as the performance in mental stress test and reaction time, while (4) Red Bull^©^ consumption did not have significant effect on subject's emotional status, precisely aggression and anxiety. Red Bull^©^ consumption significantly increased MAP and HR as well as RR and RFR, at rest and during exercise in young healthy population, all of which are under adrenergic control; it had beneficial effect on cognitive functions (immediate memory, attention, and verbal fluency), improved performance in MST, and improved RT. However, consumption of Red Bull^©^ has not altered subject's emotional status, including aggression and anxiety. 500 mL Red Bull^©^ consumption significantly increased blood glucose levels, as well as plasma adrenaline and noradrenalin levels. To our knowledge this is the first study which demonstrated that Red Bull^©^ consumption leads to adrenergic system activation with subsequent changes in both cardiovascular and psychomotoric reactions in young individuals.

We have found significantly increased DBP and MAP while SBP increased but without significance after Red Bull^©^ consumption. HR was significantly increased 30 minutes after Red Bull^©^ intake. These results are consistent with Steinke et al. who observed influence of 500 mL energy drink through 7 days and reported elevated DBP within 2 hours of energy drink consumption as well as elevated SBP and HR at day 1 of energy drink consumption [8]. A novel study from 2014 has shown that Red Bull^©^ consumption led to increases in both SBP and DBP, associated with increased HR and cardiac output, with no significant changes in total peripheral resistance and without diminished endothelial response to acetylcholine [32]. Even though some authors reported decreased HR after consuming energy drink, we found opposite changes in concordance to [4, 7, 9]. Inconsistency of the results that described the BP changes are probably caused by quantity of energy drink used in the study as well as a fasting period for caffeine before study protocol [7]. It was reported that consumption of a single can of Red Bull^©^ (250 mL) was not associated with adverse cardiovascular effects [33]. Repeated HR measurement during one-minute rest before HST, confirmed increased HR after Red Bull^©^ consumption compared to control measurement. HR was significantly increased during the first minute of exercise, as well as during the third minute of rest after exercise. This increment of arterial blood pressure and HR 30 minutes after Red Bull^©^ consumption may be associated particularly with increased sympathetic activity. Today is generally accepted that sympathetic overactivity results in increased HR and blood pressure [34, 35]. We cannot be sure which substances in Red Bull^©^ are responsible for this effect, but caffeine is a likely candidate. Although it is tempting to attribute the BP- and HR-elevating effects of Red Bull^©^ to its caffeine content and consequent sympathetic adrenal-medullar system activation, a recent pilot study [36] reported that repeated consumption of Red Bull^©^ drinks between 8:00 and 19:00 led to an increase in mean 24 h and daytime ambulatory BP when compared to caffeine consumption alone. This raises the possibility that other ingredients in Red Bull^©^, in their own rights or in interaction with caffeine, may underline elevation in BP and HR after Red Bull^©^ consumption only by adrenergic system activation and/or by involvement of another mechanism which still remains to be determined. In order to clarify this issue, it would be very important to test whether this BP- and HR-elevating effect of Red Bull^©^ would be diminished by the use of adrenergic antagonists in some future studies.

Blood glucose level significantly rose 30 minutes after Red Bull^©^ consumption. This significant increment can be attributed to intake of almost 56 g of sugars (glucose 10,5 g and saccharose 43 g) in 500 mL of Red Bull^©^. However, it has been shown that activation of adrenergic system via physical or emotional stress increases blood glucose levels (max glucose levels reached 30 minutes after stress stimulation) [37]. Furthermore, the effects of caffeine, particularly on glucose metabolism in the sedentary state are detrimental. Acute consumption of caffeine in combination with a glucose load is known to transiently impair whole-body glucose disposal and to cause hyperinsulinemia as well as hyperlipidemia. Caffeine interferes with the actions of insulin, and it is important to appreciate that this hormone affects the metabolism of lipids as well as carbohydrates [38]. With regard to carbohydrate homeostasis, caffeine containing energy drinks deliver both a readily absorbable form of glucose in combination with a dose of caffeine sufficient to impair glucose metabolism. Thus the acute and chronic influence of caffeine and sugars containing energy drinks which, according to our results, activate adrenergic system on blood glucose levels, require further careful consideration in future studies.

To our knowledge, this is the first study that measured the impact of Red Bull^©^ consumption on respiratory function at rest and during exercise. We found significantly increased RR as well as RFR at rest, during exercise and at rest after exercise. This effect of Red Bull^©^ can also be explained by activation of sympathetic nervous system, which leads to increased RT and greater alveolar oxygen exchange due to bronchioles dilation.

However, although potential link between energy drink consumption and increased sympathetic activity is accentuated in numerous studies, there is a lack of actual measurement of plasma catecholamine levels in response to Red Bull^©^ to support these speculations. That is the reason why in the present study we have measured plasma concentrations of adrenaline and noradrenalin in five different time points (before Red Bull^©^ consumption and 15, 30, 45 and 60 minutes after Red Bull^©^ consumption) and demonstrated that both adrenaline and noradrenalin plasma levels were increased after Red Bull^©^ intake through the time in which different study protocols were randomly performed compared to values before Red Bull^©^ intake (Figure 1). This is firm evidence that indeed sympathoadrenal activation is underlying mechanisms of cardiovascular and respiratory effects of RedBull^©^. As mentioned above, final confirmation of this connection between Red Bull^©^ consumption and activation of adrenergic system, would be diminished cardiovascular and respiratory effect of Red Bull^©^ consumption by adrenergic antagonist which is the natural next step in further studies.

Our finding that Red Bull^©^ intake improved RT is consistent with Alford et al. who reported that Red Bull^©^ improved reaction times, alertness and concentration, mental acuity skills that might be considered important to college students [7]. Seidl et al. have found significantly improved motor reaction after administration of caffeine-and-taurine-containing drink, similar to Red Bull^©^ [19].

As Red Bull^©^ is advertised and sold as energy drink that beside physical, improves and mental performance, it is really important to find its real effect on cognitive functions. Results of our study have shown significant improvement in working memory, verbal fluency and attention tests performance after Red Bull^©^ consumption. Immediate memory was significantly improved after consuming Red Bull^©^, when measured as number of correctly repeated numbers in forward order. In already mentioned study, Alford et al. found that Red Bull^©^ improved cognitive performance [7]. The results of Howard and Marczinski indicated that the energy drink doses decreased RTs on the behavioral control task, increased subjective ratings of stimulation and ratings of mental fatigue, suggesting improved cognitive performance [20]. Seidl et al. reported improved attention, measured with same test as in our study (d2 Attention Loading Test), after administration of caffeine-and-taurine-containing drink, similar to Red Bull^©^ [19]. Although most studies that examined the influence of energy drink on cognitive performance have shown positive effect, mechanisms are still not clear. Mechanisms for modulation of cognitive functions by the energy drink can be assigned to caffeine via its action on adrenosinergic system, which in turn is closely linked to other neurotransmitter systems [39]. Important finding in our study is a better success in MST after Red Bull^©^ consumption. Some previous studies have reported similar effect, which is contributed to the taurine, known to modulate mood as well as stress and behavioral response [40–42]. Interestingly, even though it is known that catecholamines appear to be involved in metabolic preparations for the prospective fight and that a slight activation of noradrenergic system stimulate aggression, we did not observe significant effect of Red Bull^©^ consumption with subsequent increased activity of the sympathetic nervous system on measured impulsive aggression [43, 44]. Also, we did not observe significant effect of Red Bull^©^ consumption on individual's anxiety although numerous studies have described increased sympathetic activity in patients with depression and anxiety [45], which is important in the light of increased aggressive behavior among young people. However, it should be emphasized that there has been a dramatic rise in the consumption of alcohol mixed with energy drinks in young people which has been implicated in risky drinking practices, greater accidents and injuries, and risky behavior like risky sexual behavior and violence [46, 47]. Since it is currently not entirely clarified how the combined effect of alcohol and energy drinks impact the activation and inhibition of behavior differently than alcohol would alone, more controlled laboratory studies are needed to determine if alcohol mixed with energy drinks are escalating risky drinking practices among young people.

Limitations of the study: the study was organized as before and after the treatment study, where subjects were self-controls. Due to particular taste of Red Bull^©^, it was not possible to blind subjects or examiners on the beverage taken. Because Red Bull^©^ intake obviously has important effect on glucose metabolism and blood glucose level, a glucose tolerance test would be interesting to perform, to exclude possibility that glucose is high due to intolerance and not due to high glucose content in the Red Bull^©^ to. However, as stated in Methods section, participants in this study were healthy young people with normal fasting glucose levels and no positive history of diabetes or prediabetes or glucose intolerance.

In conclusion, our study has shown consistent positive effects of Red Bull^©^ energy drink on cognitive functions (immediate memory, attention, and verbal fluency), improved performance in mental stress test and improved reaction time. These positive effects are consistent with the assertions of Red Bull^©^ manufacturers and one of the main reasons why are precisely young people and college students the main consumers of Red Bull^©^. However, results of this study have shown that 500 mL of Red Bull^©^ may have adverse effect on cardiovascular and respiratory system, leading to increased MAP and HR as well as RR and RFR, at rest and during exercise. Precisely because the target market for Red Bull^©^ is people between 15 and 30 years of age, that is, typically healthy, involved in activities, and includes a higher proportion of sports enthusiasts and high-risk takers, these effects should not be neglected. Other main problems involved in Red Bull^©^ consumption are that its numerous ingredients and its effect is difficult to assign to one specific ingredient. This is first study that has actually shown increased activity of the sympathetic nervous system (increased plasma adrenaline and noradrenalin levels) after Red Bull^©^ consumption which may have an important role in its observed effects on cardiovascular and respiratory system during rest and exercise, on cognitive functions as well as on performance in mental stress test and reaction time and thus elucidated important mechanism of its' action.

## Figures and Tables

**Table 1:** Arterial blood pressure and heart rate of study population.

Variable	Before Red Bull^©^	After Red Bull^©^	*P* value
SBP	113 ± 12	115 ± 12	0.151
DBP	68 ± 10	70 ± 8^*^	**0.03**
MAP	83 ± 10	85 ± 9^*^	**0.037**
HR	71 ± 10	76 ± 11^*^	**<0.001**

Results are expressed as mean ± SD.

SBP: systolic blood pressure, DBP: diastolic blood pressure.

MAP: mean arterial pressure, HR: heart rate.

^*^ *P* < 0.05 (statistically significant values are in bold) before Red Bull^©^ versus after Red Bull^©^ (*n* = 38).

**Table 2:** Scores achieved on tested cognitive tasks.

Cognitive function	Task	Before Red Bull^©^	After Red Bull^©^	*P* value
Immediate memory	AVLT- (1-) first trial	8,17 ± 1,77	7,79 ± 2,04	0,426
	ADST-forward	8,03 ± 0,82	8,48 ± 0,87^*^	**0,029**
Delayed recall	AVLT-delayed recall	12,58 ± 2,18	13,00 ± 1,93	0,231
Working memory	ADST-backwards	6,45 ± 0,83	6,86 ± 1,03^*^	**0,048**
Delayed verbal memory	AVLT-late recall	12,55 ± 1,97	12,03 ± 3,15	0,459
Verbal fluency	Semantic task	23,28 ± 3,89	26,10 ± 4,94^*^	**<0,001**
	Ideational task	15,07 ± 4,00	18,66 ± 4,43^*^	**<0,001**
	Phonemic task	30,70 ± 7,31	41,76 ± 9,71^*^	**<0,001**
Attention	d2 test TN-E	486,83 ± 69,19	592,45 ± 60,31^*^	**<0,001**
	d2 test MC	267,90 ± 27,90	283,21 ± 21,89^*^	**<0,001**

Results are expressed as mean ± SD.

AVLT: auditory verbal learning test; ADST: auditory digit span task; TN-E: quantitative measure defined by total number of processed data minus number of total errors; MC: mean concentration.

^*^ *P* < 0.05 (statistically significant values are in bold) before Red Bull^©^ versus after Red Bull^©^ (*n* = 27).
